# MODEM: A comprehensive approach to modelling outcome and costs impacts of interventions for dementia. Protocol paper

**DOI:** 10.1186/s12913-016-1945-x

**Published:** 2017-01-11

**Authors:** Adelina Comas-Herrera, Martin Knapp, Raphael Wittenberg, Sube Banerjee, Ann Bowling, Emily Grundy, Carol Jagger, Nicolas Farina, Daniel Lombard, Klara Lorenz, David McDaid, On behalf of the MODEM Project group

**Affiliations:** 1 Personal Social Services Research Unit, London School of Economics and Political Science, London, UK; 2 Centre for Dementia Studies at the Brighton and Sussex Medical School, University of Sussex, Falmer, Brighton UK; 3 Faculty of Health Sciences at the University of Southampton, Southampton, UK; 4 Department of Social Policy at the London School of Economics and Social Science, London, UK; 5 Institute for Ageing and Health, Newcastle University, Newcastle, UK

**Keywords:** Dementia, Costs, Outcomes, Treatments, Social care, Carers, Microsimulation model, Economics, Cost-effectiveness

## Abstract

**Background:**

The MODEM project (*A comprehensive approach to MODelling outcome and costs impacts of interventions for DEMentia*) explores how changes in arrangements for the future treatment and care of people living with dementia, and support for family and other unpaid carers, could result in better outcomes and more efficient use of resources.

**Methods:**

MODEM starts with a systematic mapping of the literature on effective and (potentially) cost-effective interventions in dementia care. Those findings, as well as data from a cohort, will then be used to model the quality of life and cost impacts of making these evidence-based interventions more widely available in England over the period from now to 2040. Modelling will use a suite of models, combining microsimulation and macrosimulation methods, modelling the costs and outcomes of care, both for an individual over the life-course from the point of dementia diagnosis, and for individuals and England as a whole in a particular year.

Project outputs will include an online *Dementia Evidence Toolkit*, making evidence summaries and a literature database available free to anyone, papers in academic journals and other written outputs, and a *MODEM Legacy Model*, which will enable local commissioners of services to apply the model to their own populations.

**Discussion:**

Modelling the effects of evidence-based cost-effective interventions and making this information widely available has the potential to improve the health and quality of life both of people with dementia and their carers, while ensuring that resources are used efficiently.

## Background

There are currently around 835,000 people in the United Kingdom (UK) who have dementia [1], and an estimated 670,000 unpaid dementia carers, most of them family members [2]. If current rates of prevalence of dementia by age and gender remain unchanged, this number will grow to more than 1 million by 2021 and 2 million by 2051 as a consequence of population ageing [1]. Even if prevalence rates are declining slightly, as some recent studies suggest [3, 4] the numbers of people living with dementia will still increase nationally and globally, where it is projected that the number will grow from 46.8 million people today, to 131.5 million in 2050 [5].

If service models remain unchanged, the costs of treatment and care for people with dementia are likely to increase more rapidly than total prevalence over the same period, since care services are highly labour-intensive and wage inflation usually runs ahead of other price increases. This will put considerable pressure on already stretched health and social care budgets and generate major increases in reliance on family carers. (We use the term ‘family carers’ in preference to ‘unpaid’ or ‘informal’ carers as neither of the latter terms is fully accurate; most carers of people with dementia in England are family members and our reference group have expressed a preference for this term.)

The MODEM project (*A comprehensive approach to MODelling outcome and costs impacts of interventions for DEMentia*) explores how changes in arrangements for the future treatment and care of people with dementia, and support for carers, could result in better outcomes and more efficient use of resources. To do this, the MODEM team is reviewing international evidence on effective and (potentially) cost-effective interventions in dementia care, and then using those findings, with analyses of existing and new cohort data, to model the quality of life and cost impacts of making these interventions more widely available in England over the period from now to 2040. The MODEM project began in 2014 and runs until February 2018. It is funded by the UK Economic and Social Research Council (ESRC) and the National Institute for Health Research (NIHR).

### Conceptual framework

Conceptually, the project is rooted in the ‘disablement process’ model and the ‘production of welfare’ framework.

The ‘disablement process’ model, proposed by Verbrugge and Jette [6], builds on previous models developed by the World Health Organization (WHO) and others. It conceptualises the ways in which needs for long‐term care arise (from pathology to impairments in specific body systems which lead to restrictions in basic physical and mental actions, and finally to disability), as well as how and at what stages individual risk factors and environmental factors might influence this process. Similarly, the International Classification of Functioning, Disability and Health (ICF) describes decreases in function as the complex and dynamic interaction of health conditions (pathology) with extra- and intra-individual factors acting to mitigate or exacerbate the process [7]. However, where the endpoint of the Verbrugge and Jette model was disablement, the ICF outline includes participation in society as the ultimate goal to attain. In addition, the more linear pathway from disease to disability in the Verbrugge and Jette model has been loosened.

At each stage of the disablement process (in this model) there are potential opportunities to halt or even reverse the process. These include altering individual risk factors for chronic conditions (e.g. through changes in lifestyle, such as smoking and exercise) or implementing improvements in the management of chronic conditions. The consequences of functional limitations associated with disability can also sometimes be reduced through aids and adaptations, information and communication technology, occupational therapy, suitable housing or relevant changes in the wider environment.

Once needs have emerged, we use the ‘production of welfare’ framework [8, 9] to capture the potential relationships between needs, resources and outcomes. This framework has underpinned much of the work of the Personal Social Services Research Unit (PSSRU) in social care, mental health and other areas over a 40-year period. The framework represents a simplification of the multifarious links between budgets, staff and other ‘inputs’ (and their associated costs), the services that are produced, the ‘non-resource’ influences on what services can achieve (such as personal resilience and staff attitudes) and the health and wellbeing outcomes that potentially result for people with dementia, their families and relevant others.

### Project aims and objectives

The MODEM project aims to generate new evidence to inform policy and practice to better and more efficiently meet needs, promote health and wellbeing for people with dementia and their family and other carers.

The project objectives are to:

1. Build a comprehensive conceptual, integrated framework that covers impacts of dementia on cognition, functioning and behaviour, responses from carers, responses from health and social care systems, the effectiveness and resource impacts of these interventions, and the potential long-term funding implications;
2. Develop a suite of linked quantitative models, employing both microsimulation and macrosimulation techniques, to project future numbers of people with dementia, their dependency and other needs, comorbidities, levels of unpaid and formal care and associated expenditure;
3. To estimate typical life-time costs of dementia, under varying assumptions about risk factors, patterns of care and support, and preferences;
4. Review the literature for evidence on interventions that could delay onset, slow deterioration in cognition, functioning or behaviour, or reduce their adverse impacts on health wellbeing, both for people with dementia and carers, and also evidence on costs;
5. Gather evidence on the lives of people with dementia and their carers by collecting primary data from a new cohort, by conducting qualitative interviews and focus groups, and by examining data from previous trials and observational studies;
6. Use the evidence from objectives 4 and 5, in combination with the micro- and macro-simulation models, to produce projections to 2040 of the numbers of older people with dementia in England, their needs for care and support, and associated public and private expenditure, together with projected outcomes and costs of a range of interventions to prevent or delay dementia incidence, slow symptom development, provide treatment and care, and support carers;
7. Develop a *Dementia Evidence Toolkit* to make available evidence summaries of the effectiveness and cost-effectiveness of dementia care and treatment interventions and a searchable bibliographic database; and
8. Develop a publicly available web-tool (*MODEM Legacy Model*) to enable service commissioners, providers, advocacy groups, individuals and families to access the findings and outputs of the project, and to make their own projections of expected needs for care and support, outcomes and costs.

## Methods

### Over-arching research strategy

We first provide an over-arching summary of the interconnected activities of the project, and then describe each of the main elements in more detail.

We are engaging with people living with dementia, carers and others at all stages of the project. We are starting by examining extant data to understand the links between the characteristics of individuals and families, their dementia-related and other needs for care and support, and the services and treatments that could be available to them. We are then looking at the effects of care, support and treatments on outcomes for individuals and carers - how those interventions can improve their health and wellbeing - and also on the costs of support.

We will use this information, as well as specially collected data from a cohort, to make projections of how many people there will be with dementia in England over the period to 2040, the family or other unpaid support they are likely to have available, and the costs to provide care services for them. Second, we are examining whether there are better ways to support people with dementia and their carers by making evidence-based forms of care and treatment more widely available. We are relying on previous evaluations of these interventions to demonstrate the effects on health and wellbeing, and on costs. We are potentially including a wide set of interventions, including medication, cognitive stimulation and other therapies, exercise programmes, nutrition advice, telecare, case management, community initiatives, respite and training for carers.

We are also collecting primary data from a new cohort 300 people with dementia and their carers at two points of time, 12 months apart. We are not testing any interventions with these people; rather, we are collecting information to allow an analysis of the relationship between measures (i.e. ‘cross-walking’) and to fill gaps in available evidence. We are additionally conducting qualitative interviews and focus groups to gain further ‘experiential’ evidence.

A range of quantitative methods will be used, including dynamic micro-simulation projection modelling, to understand the disabling consequences of dementia, and a series of care pathways models to show how evidence-based interventions can influence outcomes, service use and costs. A life-time costs model will generate estimates of the overall costs of the care pathway for each intervention, and a macro-simulation projection model will generate estimates of long-term care needs and costs to 2040.

Our final task will be to create a ‘MODEM legacy model’ to allow commissioners, providers, individuals and advocacy groups to make their own projections of needs, outcomes and costs using our estimates.

### Involving people with dementia, carers and other stakeholders

The project benefits from the regular input of key stakeholders, including members of our overarching Advisory Group, our Reference Group of people with dementia, carers and service providers, and our Impact Advisory Group (chief executives and public policy leads). The research team is building on existing links with key central government departments in England (e.g. Health, Communities and Local Government, Work and Pensions, and Treasury), third sector organisations (e.g. Alzheimer’s Society, Alzheimer’s Research UK, Carers UK), National Health Service (NHS) England and Public Health England, and local councils and their umbrella bodies (the Association of Directors of Adult Social Services, Local Government Association).

### Mapping the literature and conducting evidence reviews

A systematic mapping of relevant literature will be conducted with the aim of identifying interventions that can prevent or delay dementia onset, reduce symptom severity and/or improve quality of life of people with dementia and/or family and other carers.

We are first identifying previously published systematic reviews and meta‐analyses and then searching for papers that have not been included in previous systematic reviews (for example, because they have been published more recently). We will also identify relevant literature in areas that have not been covered by previous reviews, carrying out our own new reviews where there are gaps.

Our careful consideration of the available evidence will inform the selection of interventions to be modelled later in the project.

The review of evidence should be of value in its own right as it will identify areas in which there is strong existing evidence, as well as where there has been little research undertaken and interventions for which there is insufficient evidence. Some of the outputs from the evidence review, a coded bibliographic database and a set of evidence summaries written in non-technical language, will be made publicly available through a website, the *Dementia Evidence Toolkit*, so that people with dementia, carers, care providers, commissioners, researchers and others can access the resources.

It is expected that the review process will also draw out the implications of using different research methods for the usability of evidence in modelling and in commissioning.

### Modelling future numbers of people with dementia and their carers, costs and outcomes

The core of the project involves the development of a suite of linked quantitative models, using micro‐ and macro‐simulation, to project future numbers of people with dementia, unpaid and formal care and associated expenditure, and to estimate typical life‐time costs of dementia. The projections will be based on assumptions about risk factors, patterns of care and support, and individual preferences.

#### Macrosimulation model

We are developing a macro-simulation model to produce overall projections of future numbers of people with dementia and future expenditures on their treatment and care. It will take as inputs the outputs of the microsimulation and interventions models described below. This model builds on previous cognitive impairment and long-term care models developed at PSSRU [10, 11]. It projects future use of care and associated costs and quality of life of people with dementia and carers.

A key feature of this new model is that it will differentiate between groups of people with dementia by severity of cognitive impairment and physical disability, and it will ‘assign’ packages of care to people with dementia based on their characteristics including severity of condition and comorbidities. The model will use data from the latest MRC Cognitive Function and Ageing Study (CFAS II) [12], the English Longitudinal Study of Ageing (ELSA) [13], official data from the Health and Social Care Information Centre, the baseline data of various trials of interventions for people with dementia (see below) and new data collected as part of this study (the cohort study described above).

#### Microsimulation epidemiological model

A micro-simulation population model (MicSIMPOP) is being developed to examine the health and associated care needs of the English population over the coming decades, and the impact of interventions for risk factor reduction, disease prevention and treatments that slow down progression to disease (including dementia) and disability. This is a more comprehensive, up-to-date version of a previous macro-simulation population model (SIMPOP) [14].

The model will be based on longitudinal data from *Understanding Society* (adults 35 years and over in community dwellings [15]), ELSA (adults aged 50 years and over in community dwellings) and the new CFAS II cohort (adults 65 years and over, including those in institutions). These three datasets, suitably weighted, will allow inferences to be made for the older English population (65 years and over) to 2040. Baseline characteristics generated on these individuals will be of three types: socio-demographic (differentiated by the following variables: age, gender, living arrangements, marital status, education, retirement status); lifestyle behaviours (smoking, alcohol consumption, physical activity, body mass index, social engagement); and diseases (cognitive impairment/dementia, coronary heart disease, stroke, hypertension, diabetes, respiratory disease, arthritis, cancer) as well as geriatric conditions (hearing and vision impairment). Mortality rates from the most recent population projections will be applied by age and gender. The outcome variable will be disability measured by the interval of need scale [16], which categorises people on the basis of Activities of Daily Living/Instrumental Activities of Daily Living in terms of the intensity of care required.

The primary output will be tabulations of disability by age and gender in the presence of cognitive impairment/dementia and any other diseases which will be used as inputs to the macro-simulation framework model described earlier. The issue of co-morbidity will become crucial when determining the types of care packages required, and their cost. For other conditions, known trends in disease risk factor prevalence will be incorporated, such as smoking and obesity, as well as changes in socio-demographic variables (education, marital status, living circumstances). We will use bootstrapping to provide measures of uncertainty around estimates; the work will also include individual biographies to feed into the life-time costs model as well as calculation of disability-free life expectancy to allow exploration of the likelihood of compression or expansion of disability given different health scenarios.

#### Intervention modelling

The impact of interventions in dementia care, particularly the impacts on costs and outcomes of care for particular groups of people with dementia and for their family and other carers, will be modelled using intervention-specific models. The degree of sophistication of the models will vary depending on the nature of the impacts of different interventions and the type of evidence available on them. We envisage that some of the models may be simple decision models, while we expect to be able to carry out microsimulation analyses for other interventions. The outcomes of interventions modelling will be integrated into the macro-simulation model to produce national-level projections of changes in costs and quality of life arising from their wider adoption.

We will model only one intervention at a time, except in cases where we have evidence of combinations of interventions being evaluated together, such as maintenance cognitive stimulation therapy and donezepil [17, 18]. This is because we are not able to assess the ‘additive’ impact of different interventions unless they have already been evaluated in combination.

The choice of interventions included in this modelling will be influenced by what we find from our evidence reviews (described earlier), by what data we can access (either directly on those interventions or by parameter estimation in simulation models) and by the views of experts in the field.

#### Lifetime model

We are also developing a ‘lifetime’ model to examine the individual costs of dementia over a lifetime, and the impact on quality of life. This model considers the average duration of dementia from onset (i.e. the time at which it could first be diagnosed) to end of life. This duration is divided between periods of mild, moderate and severe dementia, and between residence in the community and in a care home. Costs of care appropriate to the severity of cognitive impairment and the type of care are attached to each month of the dementia pathway, and costs are then aggregated over the whole duration with dementia.

### Data sources

As there is no single dataset that has all the data we need for the modelling, we will bring together data from a variety of sources. As we have already described, the epidemiological model will draw upon data from three major longitudinal studies: Understanding Society, ELSA and CFAS II, this being the first time that these three studies have been combined. We also use data from ELSA and the Health Survey of England (HSE) to complement the information on use of care services and unpaid care at national level.

For our intervention models, we will carry out detailed modelling of the impact of interventions on costs and outcomes using individual-level baseline or ‘usual care’ group data from a number of recently completed or ongoing dementia trials in the UK (details available on request). In this way we can understand the relationships between particular patterns of needs (e.g. cognitive impairment, behaviour, functional disability), individual characteristics (e.g. age, gender, co‐morbidities) and circumstances (e.g. living alone, socioeconomic status) of the person with dementia and their carers.

#### Primary data collection

Since our models will combine data from various population surveys, clinical trials and observational studies, we need to be able to combine and compare different measures of the same underlying domains (e.g. cognition, carer impact or quality of life). We are able to use trial’ data to explore associations between two or more different measures of the same domain, but these only provide some of the information we need. We are therefore collecting primary data from a cohort of people with dementia and carers.

In face-to-face interviews, we ask 300 dyads of people with a diagnosis of dementia and their carers to complete a selection of overlapping measures of need, care use and outcomes. Analysis of the resulting data enables us to ‘cross-walk’ between the different measures and studies. In addition the dataset will provide valuable information in its own right. For example, we use an adapted version of the Client Service Receipt Inventory (CSRI) [19] so that, as well as gathering information on use of services by people with dementia and support from family and other carers, evidence can be built up on some key associations (such as carer age and gender, and their links to carer wellbeing).

Individuals included in this cohort study have been identified from the clinical populations served by the Sussex Partnership NHS Foundation Trust with support for recruitment through the Join Dementia Research initiative funded by the Department of Health and delivered in partnership with the National Institute for Health Research, Alzheimer Scotland, Alzheimer’s Research UK and the Alzheimer’s Society. Participants are drawn from East and West Sussex and Brighton and Hove, a population of 500,000 older adults, which includes an estimated 30,000 people with dementia; similar to the national population except with respect to deprivation and ethnicity (although it is unlikely this will have a major bearing on the cross-walking of parameters). People with dementia involved in the study all have a clinical diagnosis of dementia, established using ICD‐10 criteria. The cohort is being stratified by dementia severity, with 100 people with mild dementia (i.e. scoring 20+ on the standardised Mini‐Mental State Examination (sMMSE) [20]), 100 people with moderate dementia (scoring 10–19) and 100 people with severe dementia (scoring 0–9). A sample of 300 subjects gives sufficient precision to generate the insights we need into relationships between variables.

People with dementia and their carers are being interviewed at baseline and 52 weeks later in either the clinic that they usually attend or in their own home. Interviews are designed to minimise respondent burden while still collecting comprehensive data. Data collection is split between people with dementia and carers, who are interviewed simultaneously by researchers operating in pairs. Individuals are sent a written invitation to take part in the study by their clinical team; this is followed up by telephone contact to arrange a home or clinic visit. At the first meeting the researchers assess capacity and obtain appropriate consents; if consent is given, the people with dementia and their main family carer are interviewed.

#### Analysing relationships between characteristics, needs, resources and outcomes

A key part of the project involves gaining a better understanding of the relationship between the individual characteristics and circumstances of both people with dementia and unpaid carers, their needs, resources, and how these relationships are likely to affect the outcomes of interventions. We are using both quantitative and qualitative methods to understand these relationships, which will provide the key parameters for the models. We are focussing in particular on the following areas.

##### Relationships between personal characteristics, need factors, use of paid and unpaid care and quality of life

Using as a framework the ‘production of welfare’ approach, we are analysing a number of datasets to understand better the relationships between personal characteristics such as age, gender, household type, education, severity and type of care needs, the use of family and paid care, and the quality of life of people with dementia and their carers. Multivariate analyses will provide us with parameters that will be used in the simulation models (see above).

##### Social interaction and participation over the life‐course

Factors associated with cognitive ability and dementia include age, gender, education, socioeconomic status, and smoking [21]. There is also growing awareness of the protective or buffering effect of social participation, while indicators of social isolation are risk factors for cognitive decline [22, 23]. This may reflect both effects of social interaction on cognition and effects of social resources on coping strategies adopted in the face of impairment, as proposed in the theoretical models of selection, optimisation and compensation [24]. As children are an important source of social support for older people, we have investigated whether fertility histories are associated with social participation and with cognitive function in later life [25]. Results suggest disadvantages for childless older people, even after taking into account socio-economic status, health-related behaviours and social contacts. We are undertaking further work on possible direct or indirect effects of long-term social interaction on cognitive functioning in early and later old age. We are also examining the effect of accumulated social support networks on formal help-seeking and receipt of services, among those with cognitive impairment.

‘Cognitive reserve’ is a widely used construct to explain how, in the face of neurodegenerative changes similar in nature and extent, individuals vary in their severity of cognitive ageing and clinical dementia [26]. People with high reserves may have increased capacity for continued learning and adaptation, despite age-related changes. Data on cognitive functioning are taken from the National Child Development Study (NCDS) and ELSA [27]; both used the same measure at key waves (age 50 for NCDS (and educational performance indicators up to age 16); ages 50 and above from Waves 1–5 of ELSA). They also include measures of social networks, support and, in the case of ELSA, service use. Social interaction is defined in terms of contacts with social network members, and includes size, type, and support; social participation is defined by engagement in social and leisure activities, including physical activity.

##### The wellbeing of family carers: investigating gender and relational differences

A PhD studentship attached to MODEM (held by KL) is investigating the influence of age and gender on the wellbeing of male and female family carers of people with dementia, distinguishing those of the same and next generation. The two core research questions are:

- Are there measurable differences in the wellbeing of male and female family carers for people with dementia of the same and next generation?
- Does the wellbeing of unpaid carers for people with dementia differ from the general British population?

To answer this research question, data from three sources will be pooled and analysed: the StrAtegies for RelaTives study (START) [28], the Support at Home: Interventions to Enhance Life in Dementia: Carer Supporter Programme — Remembering Yesterday Caring Today (SHIELD CSP RYCT) [29] and data from the MODEM cohort described above. All three studies share important variables on carer characteristics, standardised measures for carer wellbeing, as well as some important variables related to the person with dementia.

The second part of this PhD work focuses on the qualitative aspects of how men and women of different ages experience the provision of unpaid dementia care and construct wellbeing. In-depth interviews are being conducted with participants of the MODEM cohort study. Thematic analysis will be used to analyse the data.

The third part investigates the costs incurred by family carers. For this an amended form of the Resource Utilization in Dementia (RUD) instrument [30] is being collected as part of the MODEM cohort study. The study aims to analyse whether there are differences in costs by gender and relationship to the care recipient.

##### Understanding the experiences of people with dementia and their carers

Interactive focus group settings will provide opportunities for people with dementia and family carers to discuss their attitudes, beliefs and experiences with others. The discussions will be based on issues emerging from the modelling and cohort survey, with potential participants to be recruited through voluntary sector organisations for people with dementia and carers, and the team’s existing contacts. The groups will help provide insights and understandings for interpreting quantitative findings, including carers’ experiences of the processes of accessing dementia‐related services.

Group discussions have potential advantages over individual interviews for people with cognitive impairments, including enhanced quality of interaction, reduced pressure on individuals to respond, mutual support, and that shared experiences can trigger ideas and memories.

Using skilled facilitators with experience of working with people with dementia has been found to be important, especially in giving prompts to move people on to new areas of discussion and to avoid leading anyone with answers.

We will set up four focus groups of eight people each with dementia of mild severity, and four separate groups of eight carers each (held at same venues, at the same times), at key points within the project to aid interpretation of modelling and quantitative analyses.

#### MODEM Legacy model

The final objective of the study is to develop a legacy model, to enable stakeholders to access the findings and outputs of the project, and make their own local projections of expected outcomes and costs by entering data relevant to local needs. This will be publicly available on the World Wide Web, and is aimed at service commissioners, policy makers, providers, advocacy groups, and individuals and families affected by dementia. It will enable high-level planning of services and will allow commissioners and providers to explore the implications of demand for services and associated costs, based on varying assumptions of prevalence rates of dementia in the future and patterns of care. It will be user‐friendly, with an easy‐to operate ‘front end’ and accessible user guide. We will consult people with dementia, family carers and representatives of the NHS, local authorities and voluntary sector organisations in our planning of the legacy model.

## Discussion

Dementia has enormous impacts on the health and quality of life for people with the condition, their families and other people who care for them. Many people with dementia need care in many areas of their lives, and use a range of health and social care services, as well as getting support from their family carers. As the symptoms of dementia worsen, some people will need to move into care homes. The costs of care and support can therefore be high. As the English population ages over the coming decades, so the number of people with dementia will increase considerably. This poses a potentially major challenge for health and care systems that are already very stretched: how can we ensure a good quality of life for people with dementia and their carers at a cost that is considered by society to be affordable?

The MODEM project is using a range of interconnected methods to feed new evidence into this national debate about how to respond to the challenge of dementia. We are developing a comprehensive, integrated set of quantitative models to estimate current and future needs, and the outcomes and costs of interventions aimed at meeting them, taking into account the complexity of individuals’ lives. We are collecting rich qualitative data to help us interpret the associations in those models. And we are then simulating the impacts of interventions for which there is robust or promising evidence from completed or on-going trials or other studies.

## Abbreviations

CFAS II: Cognitive function and ageing study
CSRI: Client service receipt inventory
ELSA: English longitudinal study of ageing
ESRC: Economic and social research council
HSE: Health survey of england
ICF: International classification of functioning, disability and health
MicSIMPOP: Micro-SIMulation POPulation model
MODEM: MODelling outcome and costs impacts of interventions for DEMentia.
NCDS: National child development study
NHS: National health service
NIHR: National institute for health research
PSSRU: Personal social services research unit
RUD: Resource utilization in dementia
SHIELD CSP RYCT: Carer supporter programme — remembering yesterday caring today
SIMPOP: Macro-SIMulation POPulation model
sMMSE: Standardised mini‐mental state examination
START: StrAtegies for RelaTives study
UK: United Kingdom
WHO: World Health Organization
